# *Clostridium tetani* Osteitis without Tetanus

**DOI:** 10.3201/eid2009.131579

**Published:** 2014-09

**Authors:** Pierre-Yves Levy, Pierre-Edouard Fournier, Laurène Lotte, Matthieu Million, Philippe Brouqui, Didier Raoult

**Affiliations:** Centre Hospitalier Universitaire de La Timone, Marseille, France (P.-Y. Levy, P.-E. Fournier, L. Lotte, D. Raoult);; Aix-Marseille Université, Marseille (P.Y. Levy, P.-E. Fournier, M. Million, P. Brouqui, D. Raoult);; Marseille Teaching Hospitals, Marseille (P.-Y. Levy, P.-E. Fournier);; Hôpital Nord, Marseille (P. Brouqui)

**Keywords:** *Clostridium tetani*, osteitis, bacteria

**To the Editor:** Posttraumatic osteoarticular infections caused by *Clostridium* spp. are rare, and their outcomes are often unfavorable because of the persistence of the bacteria in bone (*1*,*2*). In a recent series of 12 patients (*2*), only 1 case of posttraumatic osteoarticular infection was caused by *C. tetani* (fracture of the distal humerus with polymicrobial infection). However, no information was available about the production of tetanospasmin by the infecting strain. 

To the best of our knowledge, the only case of *C. tetani* infection with a toxigenic strain but without tetanus or osteitis was a wound infection that quickly improved after administration of antitetanus vaccine, prophylactic immunoglobulins, flucloxacillin, and metronidazole (*3*). The absence of clinical signs of tetanus despite chronic *C. tetani* infection probably resulted from vaccine-induced immunity and the fact that the patient received a booster vaccination and prophylactic immunoglobulins as soon as *C. tetani* had been identified. Retrospective immunochromatic testing of the patient’s serum seemed to confirm this hypothesis. We report a case of osteitis caused by *C. tetani* in which clinical signs of tetanus did not develop despite production of tetanospasmin by the infecting strain.

In August 2011, a 26-year-old man was admitted to Nord Hospital in Marseille, France, because of an open fracture of his left tibia and fibula, contaminated with soil. The patient had been vaccinated against tetanus in 1997 and worked in scraps recycling, He rapidly underwent osteosynthesis (locking plates). Despite receiving oral amoxicillin–clavulanate (1 g 2 times/day) for 7 days, he was readmitted 12 days later for fever and suppuration of the leg wound and underwent a second surgical debridement. A bone biopsy sample revealed *Enterococcus faecalis*, *Enterobacter cloacae*, and *C. tetani*. Identification of *C. tetani* was confirmed by 16S rRNA amplification and sequencing (99.8% identity to *C. tetani,* GenBank accession no. AE015927). The organism was susceptible to amoxicillin, rifampin, vancomycin, and metronidazole. Because antitetanus vaccine had not been administered at the time of his previous hospital admission, a dose of vaccine and prophylactic immunoglobulins were administered at this time. Treatment with intravenous imipenem (1 g 3 times/day) plus oral ciprofloxacin (500 mg 3 times/day) was initiated for 1 month, followed by oral amoxicillin–clavulanate (1 g 2 times/day) plus ciprofloxacin (500 mg 3 times/day) for 1 month and then oral amoxicillin (2 g 3 times/day) for 2 months. 

In February 2012, because bone consolidation had not occurred, the patient underwent surgical revision to remove the locking plate, clean the wound, and insert an external fixator. Cultures of specimens collected during surgery were negative. Serologic qualitative immunochromatic test result was positive for *C. tetani*. The patient received intravenous vancomycin and imipenem (1 g 2 times/day each) for 1 month, followed by oral amoxicillin (3 g 2 times/day), rifampin (300 mg 3 times/day), and ciprofloxacin (500 mg 3 times/day) for 3 months.

 In July 2012 (11 months after the accident), because of fistula persistence, the patient underwent ablation of a tibial sequestrum (Figure) and implantation of a temporary cement spacer containing gentamicin and vancomycin. The only bacterium isolated from a tibial biopsy sample was *C. tetani*. 

**Figure:**
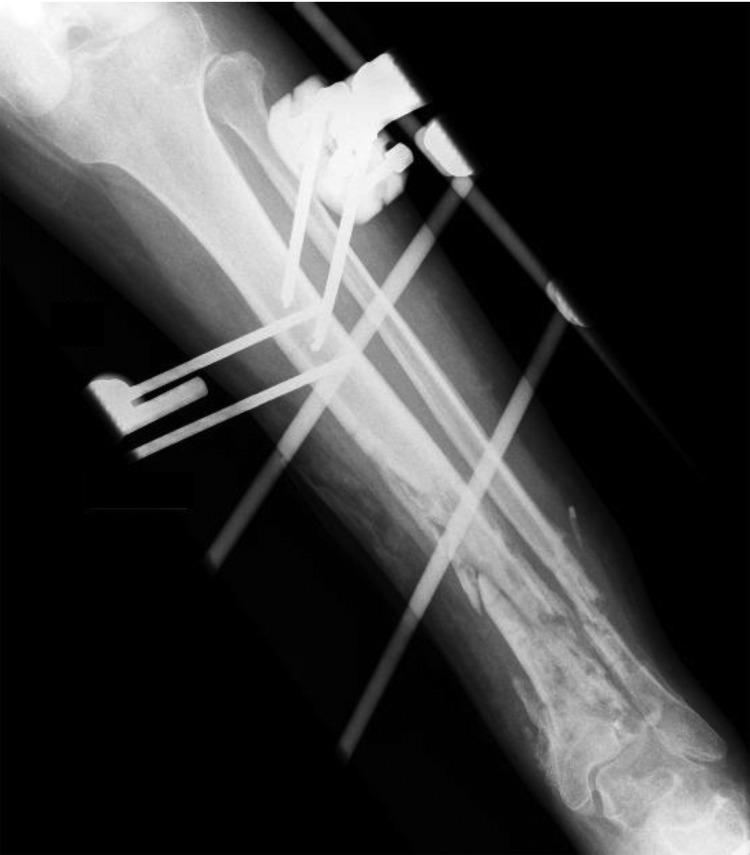
Radiograph of left leg of patient with *Clostridium tetani* infection, showing delayed bone consolidation 11 months after fracture.

The causative strain was referred to the Centre National de Référence des Bactéries Anaérobies et du Botulisme, Pasteur Institute, Paris, where presence of the *tetX* gene encoding the tetanus neurotoxin was confirmed. Oral treatment with clindamycin (2.4 g/day) for 4 months was prescribed. However, because of the unfavorable outcome despite multiple interventions and antimicrobial drug regimens, the left leg was amputated 17 months after the accident.

The case reported here is remarkable because clinical tetanus did not develop despite the production of tetanospasmin by the infecting strain and because late relapse occurred despite adapted treatment. The persistence of infection might be explained by a questionable initial antimicrobial drug regimen but also by spore formation and/or poor diffusion of antimicrobial drugs, as suggested by the presence of necrotic tissues such as the bone sequestrum. However, surgical revision, notably the ablation of this defect, should have facilitated the recovery and decreased bacterial concentration. In the literature, 3 cases of relapsing *C. tetani* infections have been reported, but those patients had not received antitetanus vaccine and they did show signs of tetanus (*4*,*5*); 1 of these patients with mandible necrosis experienced relapse 8 months after discontinuation of metronidazole. 

The pathogenesis of *C. tetani* has mainly been attributed to its toxin. Our report suggests that *C. tetani* can also cause focal infections, notably severe chronic osteitis after open fractures, especially because the anatoxin-based antitetanus vaccine does not prevent colonization and infection.
